# A Loading Dose of IV Salbutamol in an Adolescent with Severe Acute Asthma and Cardiac Arrest

**DOI:** 10.1155/2019/5057390

**Published:** 2019-09-09

**Authors:** Shelley A. Boeschoten, Ruben S. van der Crabben, Annemie L. M. Boehmer, Matthijs de Hoog, Corinne M. P. Buysse

**Affiliations:** ^1^Department of Pediatric Intensive Care, Erasmus Medical Center, Sophia Children's Hospital, Rotterdam, Netherlands; ^2^Department of Anesthesiology, Erasmus Medical Center, Rotterdam, Netherlands; ^3^Helicopter Emergency Medical Service, Erasmus University Medical Center, Rotterdam, Netherlands; ^4^Department of Pediatric Pulmonology, Erasmus Medical Center, Sophia Children's Hospital, Rotterdam, Netherlands

## Abstract

Severe acute asthma (SAA) can lead to respiratory failure and can be fatal. For rational use of intravenous (IV) bronchodilators, evidence regarding the pharmacokinetics and pharmacodynamics is lacking in children. The use of a loading dose IV salbutamol is not mentioned in any international guideline, and its use varies greatly between PICUs worldwide. We describe a 17-year-old Caucasian female with SAA resulting in an out-of-hospital cardiac arrest. After basic life support and return of spontaneous circulation, the ambulance administered oxygen, inhaled salbutamol, IV magnesium sulphate, and systemic corticosteroids. Despite of this, she was still in severe respiratory distress. Therefore, a loading dose of IV salbutamol was administered, after which an immediate improvement was observed. Having a loading dose of IV salbutamol available for emergency medical services use for SAA in children with life-threatening SAA in the out-of-hospital setting is important to consider. Further study on the dose and the effect of a loading dose IV salbutamol in children with SAA is necessary.

## 1. Introduction

Severe acute asthma (SAA) is defined as an acute asthma exacerbation that does not respond to conventional treatment with bronchodilators and systemic corticosteroids. SAA potentially progresses to respiratory failure and can be fatal [1]. In the Netherlands, approximately 100 patients are admitted every year to a pediatric intensive care unit (PICU) with an increasing incidence over the last decade [2]. Inhaled beta-agonists combined with steroid treatment are the mainstay of SAA treatment. If the patient does not respond to initial inhaled therapy there are several therapeutic options which are not standardized over PICUs worldwide. Intravenous (IV) salbutamol in children is an optional treatment with a lack of evidence regarding the pharmacokinetics (PK) and pharmacodynamics (PD) [3].

## 2. Case

**Figure 1:**
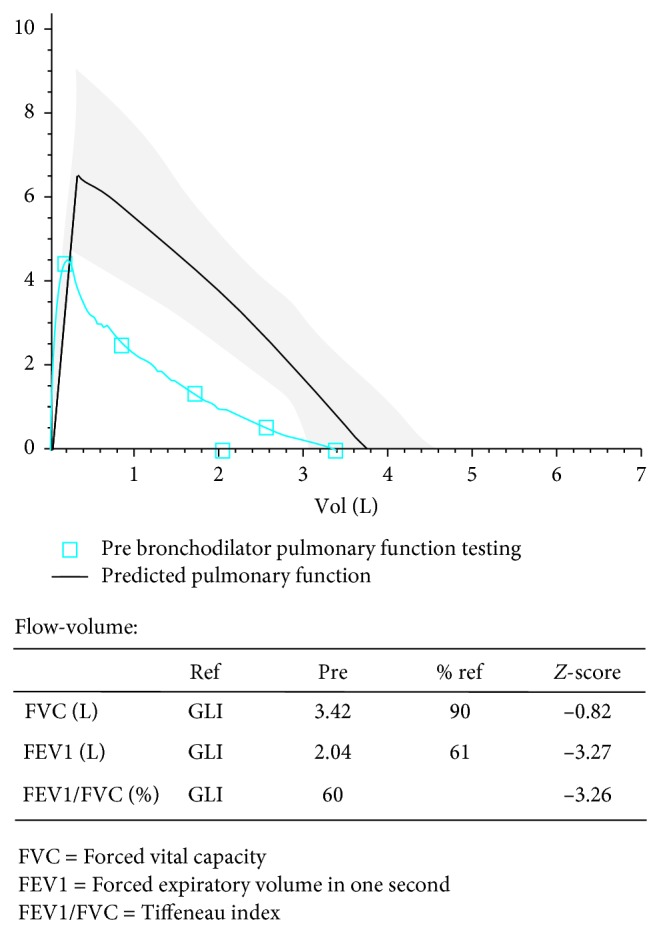
Lung function two weeks prior to the event.

A 17-year-old Caucasian female presented with a known history of severe asthma with multiple hospital admissions due to asthma in the past (not requiring admission to the PICU). She had a history of allergies for pets, tree pollen, grass, and house dust mite for which she used an oral antihistamine (desloratadine). There were no pets at home and no carpets. Recently, she started smoking again. Maintenance treatment consisted of a combination of inhaled corticosteroids with a long-acting beta_2_-agonist (beclomethasone/formoterol, 200/12 mcg twice daily) and a short-acting beta_2_-agonist as needed (400 mcg of salbutamol). There was a lack of medication adherence, as reported by herself and her parents. Pulmonary function testing two weeks prior to her cardiac arrest demonstrated severe obstruction (Figure 1).

In the weeks prior to the event, she complained of increasing shortness of breath and used salbutamol more frequently. At a scheduled visit to the pediatric pulmonologist, three weeks before the event, antibiotics and an oral course of prednisone for two weeks were prescribed for an asthma exacerbation. The symptoms did not fully disappear. A week after she finished the prednisone course, she woke up in the middle of the night because of respiratory distress and started with nebulization with salbutamol every 3 hours after her parents had consulted the emergency department (ED) by phone. In the daytime she was feeling better. The following night, again, she was in severe respiratory distress. She subsequently collapsed in front of her parents. Her parents noticed that her skin and lips turned blue, she stopped breathing, and there were no signs of life. Basic life support (BLS) was started by her father, for approximately 6 minutes.

On arrival of the ambulance there was return of spontaneous circulation (ROSC). Her oxygen saturation level was 74% in room air, with a heart rate of 180 beats per minute (bpm). She looked grey and was unresponsive. Treatment consisted of oxygen (with a nonrebreather mask (NRM)), continuous inhalation with salbutamol (15 mg/hr) and anticholinergics (ipratropium bromide, 4 consecutive doses of 0.5 mg), and IV hydrocortisone (200 mg). At the time of mobile medical team (MMT) arrival (T10 minutes), she had a persistently increased work of breathing with oxygen saturation levels of 90% with a NRM with a flow of 6 L/min. There was sinus tachycardia, with a heart rate of 150 bpm and a blood pressure of 160/100 mmHg. She was very agitated, unable to speak more than 1-2 words, and had a reduced level of consciousness. On auscultation, there were diminished breath sounds with wheezing during a prolonged expiratory phase. IV magnesium sulphate (MgSO_4_) (2 grams) was administered (based on an estimated body weight of 60 kg), and continuous nebulization with salbutamol and ipratropium bromide was continued. Because severe respiratory distress persisted, a loading dose of IV salbutamol (5 mcg/kg, 300 mcg) was given over approximately 10 minutes. After this loading dose of IV salbutamol an immediate improvement was seen by the MMT. Her oxygen saturation level increased from 90% to 95%, and the heart rate decreased from 150 bpm to 110 bpm with a blood pressure of 140/90 mmHg. She was able to speak full sentences and had a normal level of consciousness. She was transferred to the hospital with oxygen supply (NRM) and continuous nebulization with salbutamol and ipratropium bromide.

**Figure 2:**
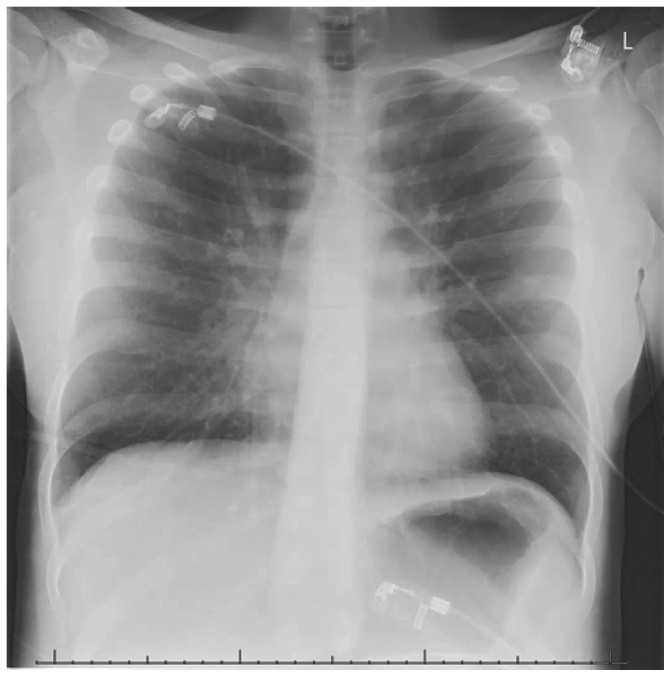
X-ray PICU admission.

At the time of arrival in the ED (T60 minutes) her vital parameters were normal (Table 1). She was able to speak full sentences. On auscultation, there were mild diminished breath sounds with wheezing end-expiratory. A capillary blood gas showed a pH 7.29, PCO_2_ 5.7 kPa (42.8 mmHg), PO_2_ 5.6 kPa (42 mmHg), bicarbonate 20.7 mmol/L, base excess –6, lactate 3.6 mmol/L, and a glucose level of 13.1 mmol/L (236 mg/dL). The X-ray showed hyperinflation and prominence of the hilar vasculature with a focal atelectasis in the middle lobe (Figure 2). Treatment at the ED consisted of oxygen supply, continuous salbutamol nebulization, and prednisone. Because there were no signs of respiratory distress anymore, no continuous infusion with salbutamol was started. Due to the severity of the SAA with the need for cardiopulmonary resuscitation, she was admitted to the PICU with continuation of the same treatment (oxygen, nebulization with salbutamol, and systemic corticosteroids). With 2 L/min of low-flow oxygen via a nasal cannula her oxygen saturations remained >95%.

Five hours after PICU admission, she was switched to hourly nebulization. The following day, she was transferred to the general pediatric ward with nebulization every two hours. Oxygen supply was discontinued after 48 hours. After 5 days, she was discharged home with a course of oral prednisone (15 days in total), 3 inhalations of beclomethasone/formoterol twice daily, and 4 inhalations of salbutamol four times daily.

One week after hospital discharge, she was able to perform all normal daily activities.

## 3. Discussion

This case report is illustrative for the potential benefit of a loading dose of IV salbutamol in children with life-threatening SAA in the out-of-hospital setting. This patient developed a SAA with respiratory failure most likely based on medication nonadherence combined with smoking. Within minutes, there was a witnessed cardiac arrest probably due to hypoxemia. Bystander BLS led to ROSC. There was a lack of response to conventional treatment, but an immediate and sustained response of her vital parameters and overall clinical picture to a loading dose of IV salbutamol.

Severe bronchial constriction may decrease the delivery of inhaled beta-agonists to the distal airways in children with SAA. Therefore, it is likely that a loading dose of IV salbutamol can lead to therapeutic concentration of salbutamol in blood immediately after infusion and thus, a more effective bronchodilation effect. The optimal dosage of a loading dose of IV salbutamol is yet to be discovered. Following 2 current national guidelines [4, 5], children receive a 10–15 mcg/kg loading dose over 5–10 min, with a maximum of 250 mcg. The loading dose advice is based on a few studies looking at the effect of a bolus of IV salbutamol [6, 7] or terbutaline [8] of 10–15 mcg/kg on asthma scores and hospital admission, showing a small effect on time to discharge from hospital or PICU, duration of nebulization of salbutamol, and clinical asthma scores. Furthermore, there were no statistical differences in side effects. Our patient received a loading dose of IV salbutamol of 5 mcg/kg (300 mcg). Although this is not high on a per kg basis, it is above the recommended maximum dose. In our patient, this lower dose led to a significant effect. Salbutamol exists as a pair of enantiomers, (R)- and (S)-enantiomer. The (R)-enantiomer is a potent B_2_-adrenoceptor stimulant, with little or no activity from the (S)-enantiomer. R-salbutamol is metabolized up to 12 times faster than S-salbutamol, which leads to relatively higher plasma concentrations of S-salbutamol [9].

Interestingly, the patient's heart rate decreased after continuous inhalation with salbutamol and even further, after a loading dose of IV salbutamol (from 180 bpm to 100 bpm), the opposite effect to what one would expect with the use of salbutamol. This could be mainly explained by the dramatic improvement of respiratory distress, as often seen in our clinical practice. Although our patient did not receive continuous infusion of salbutamol, a continuous IV infusion of salbutamol after the loading dose is recommended. Although not studied in the literature to date, it is likely to maintain therapeutic concentrations of the drug in blood at least during the transportation to the PICU. The use of adjunct therapies in the treatment of children with SAA is not standardized and varies greatly worldwide [10, 11]. Adjunct therapies which are often used are MgSO_4_, methylxanthines (e.g., theophylline), and a loading dose of IV salbutamol, even though these therapies show inconclusive results [3, 12]. In a recent survey, IV salbutamol loading dose was used by 18% of respondents from European PICUs, so it is not standard of care (under revision). Although there are many ways to treat a child with SAA, we showed that in an out-of-hospital setting a loading dose of IV salbutamol can be beneficial. Having salbutamol available for emergency medical services (EMS) use for SAA may be important to consider. Salbutamol is easy to administer in the out-of-hospital setting with very few serious side effects. Common adverse reactions associated with salbutamol therapy include tremor, hypokalaemia, increased lactate level, hyperglycaemia, and sinus tachycardia [3]. In adults, salbutamol toxicity is associated with blood concentrations >30 ng/mL with a putative lethal level of >160 ng/ml. However, in children very high blood salbutamol concentrations (196–586 ng/mL) have been recorded without serious side effects [3]. Further research is needed to evaluate the effect and the dosage of a loading dose of IV salbutamol in children.

## 4. Conclusion

In children with life-threatening SAA in the out-of-hospital setting a loading dose of IV salbutamol should be considered. Further study on the dose and the effect of a loading dose IV salbutamol in children with SAA is necessary.

## Figures and Tables

**Table 1:** Vital parameters.

	Arrival ambulance (T0)	Arrival MMT (T10 minutes)	Departure MMT (T40 minutes)	Arrival ED (T60 minutes)
Work of breathing	Increased work of breathing	Increased work of breathing	—	Normal
Oxygen saturation (%)	74	90	95	99
Oxygen supply	—	6 L/min 100%	6 L/min 100%	6 L/min 100%
HF (min)	180	150	110	100
Blood pressure (mmHg)	—	160/100	140/90	130/80
Level of consciousness		Agitated Not able to speak more than 1-2 words		Normal Able to speak full sentences
